# Poro-viscoelastic material parameter identification of brain tissue-mimicking hydrogels

**DOI:** 10.3389/fbioe.2023.1143304

**Published:** 2023-04-10

**Authors:** Manuel P. Kainz, Alexander Greiner, Jan Hinrichsen, Dagmar Kolb, Ester Comellas, Paul Steinmann, Silvia Budday, Michele Terzano, Gerhard A. Holzapfel

**Affiliations:** ^1^ Institute of Biomechanics, Graz University of Technology, Graz, Austria; ^2^ Department Mechanical Engineering, Institute of Applied Mechanics, Friedrich Alexander-University Erlangen-Nürnberg, Erlangen, Germany; ^3^ Center for Medical Research, Gottfried Schatz Research Center, Core Facility Ultrastructure Analysis, Medical University of Graz, Graz, Austria; ^4^ Division of Cell Biology, Histology and Embryology, Gottfried Schatz Research Center, Medical University of Graz, Graz, Austria; ^5^ Department of Physics, Serra Húnter Fellow, Universitat Politècnica de Catalunya (UPC), Barcelona, Spain; ^6^ Glasgow Computational Engineering Centre, University of Glasgow, Glasgow, United Kingdom; ^7^ Department of Structural Engineering, Norwegian University of Science and Technology (NTNU), Trondheim, Norway

**Keywords:** brain tissue, hydrogel, polyvinyl alcohol, biomechanical testing, indentation, parameter identification, poroelasticity, viscoelasticity

## Abstract

Understanding and characterizing the mechanical and structural properties of brain tissue is essential for developing and calibrating reliable material models. Based on the Theory of Porous Media, a novel nonlinear poro-viscoelastic computational model was recently proposed to describe the mechanical response of the tissue under different loading conditions. The model contains parameters related to the time-dependent behavior arising from both the viscoelastic relaxation of the solid matrix and its interaction with the fluid phase. This study focuses on the characterization of these parameters through indentation experiments on a tailor-made polyvinyl alcohol-based hydrogel mimicking brain tissue. The material behavior is adjusted to *ex vivo* porcine brain tissue. An inverse parameter identification scheme using a trust region reflective algorithm is introduced and applied to match experimental data obtained from the indentation with the proposed computational model. By minimizing the error between experimental values and finite element simulation results, the optimal constitutive model parameters of the brain tissue-mimicking hydrogel are extracted. Finally, the model is validated using the derived material parameters in a finite element simulation.

## 1 Introduction

The brain is one of the most important and complex organs in the human body (Budday et al., 2020a). Its structure is highly heterogeneous, with fairly dense gray matter regions containing mainly neuronal cell bodies and fibrous white matter regions characterized by axons, astrocytes and microglial cells. Fluid occupies a large fraction of the brain volume, partly stored in cells and partly as free-flowing interstitial fluid (Hladky and Barrand, 2014). At the macroscopic scale, the mechanical behavior of human brain is characterized by a super-soft, highly nonlinear material response with a remarkable time-dependence (Goriely et al., 2015). Fluid-related effects control the biomechanics of brain tissue in the healthy as well as in the pathological state of the human brain (Franceschini et al., 2006; Budday et al., 2017a; Forte et al., 2018). In addition, flow-independent viscoelastic effects dominate the short-term response of the brain, particularly relevant during impact loading, such as traumatic brain injury (Waxweiler et al., 1995).

From an experimental perspective, characterizing the mechanical behavior of the human brain is an extremely delicate task, and experimental data reported in the literature are often contradictory (Budday et al., 2017a; Faber et al., 2022). There are also limited availability, costs and ethical aspects to be considered. For this reason, synthetic surrogates and mimicking materials are increasingly being used. Among a variety of solutions that have been studied and developed, hydrogels appear to be the ideal candidate to mimic the mechanical behavior of soft hydrated tissues, including but not limited to brain tissue (Forte et al., 2016; Navarro-Lozoya et al., 2019; Axpe et al., 2020; Distler et al., 2020; Vanina et al., 2022). Used as phantoms, they provide the opportunity to mimic the mechanical behavior of native biological tissues for a variety of applications such as the development of real-scale training models for surgeons, testing of robotic surgical tools, as well as design of prostheses and personal protective gear (Fan and Gong, 2020). Additionally, when the constituent polymer is biocompatible, hydrogels find applications in drug delivery (Li and Mooney, 2016) and tissue engineering (Lee and Mooney, 2001; Peppas et al., 2006; Hoffman, 2012)—an area greatly stimulated by the rapid advances in additive manufacturing techniques (Tan et al., 2017; Heinrich et al., 2019). Several formulations of mimicking materials have been proposed for biomedical applications (Zhang and Huang, 2021), including hydrogels obtained from synthetic polymers, such as polyvinyl alcohol (PVA) based gels (Liu and Ovaert, 2011; Baker et al., 2012; Kumar and Han, 2017), and hydrogels derived from biopolymers (Wang and Spector, 2009; Distler et al., 2020). In particular, the PVA-based hydrogels (Chatelin et al., 2014; Mackle et al., 2020; Todros et al., 2022) and composites with Phytagel (PHY) (Forte et al., 2016; Leibinger et al., 2016; Tan et al., 2018; Statnik et al., 2020; Tejo-Otero et al., 2022) have shown promising potential to match and investigate the mechanical behavior of biological tissues.

Hydrogels are polymeric networks swollen with a liquid, ranging from ten up to thousands of times their dry weight. From a mechanical point of view, they essentially behave like very soft rubbers at short time scales. At longer time scales, gels undergo large volumetric changes, losing or absorbing water in response to the applied stress. At the molecular level, the origin of this mechanical behavior is well-known. Hydrogels consist of long cross-linked chains that form an entangled network and smaller solvent molecules that can move around (Hong et al., 2008). The elastic response arises primarily from the variation in entropy of the polymeric chains as the material is stretched. The time-dependent response originates from the long-range motion of the fluid molecules, i.e., the migration of the solvent into the network. In addition, viscoelastic relaxation occurs in physical hydrogels, i.e., hydrogels in which weak molecular entanglements provide cross-links that can be disrupted by changes in physical conditions (Zhao et al., 2010).

While the distinction between viscoelastic and fluid-related relaxation is clear at the molecular level, this issue is far from resolved in the context of experimental procedures. From a physical point of view, the effective diffusivity of the solvent characterizes the fluid relaxation, so that the associated relaxation time depends on a size parameter (Hu and Suo, 2012). In contrary, the process of viscoelastic relaxation is independent of a length scale as long as the mesh size of the network is much smaller than the characteristic size parameter (Hu et al., 2010). Taking advantage of this observation, several groups have attempted to separate the concurring relaxations by controlling the time and/or length scales of the experiments. In particular, indentation has been widely used in connection with hydrogels (Olberding and Suh, 2006; Galli et al., 2009; Hu et al., 2010; Kalcioglu et al., 2012; Wang et al., 2014) and in the characterization of animal and human brain tissue (van Dommelen et al., 2010; Budday et al., 2015; Feng et al., 2017; Qian et al., 2018; Jamal et al., 2022; Su et al., 2023).

A detailed understanding of the mechanical behavior of the brain is essential for the development of accurate constitutive models. In particular, the poro-viscoelastic nature of brain tissue, the interaction between fluid-related and viscous relaxation, and the interpretation of such effects from experimental observations remain only partially understood (Greiner et al., 2021). In this work, we take advantage of the controllable microstructure and reproducibility of a brain tissue-mimicking hydrogel to gain additional insight into the poro-viscoelasticity of soft hydrated tissues. We perform indentation experiments on hydrogel samples and on *ex vivo* porcine brain tissue to show the mimicking behavior related to the relaxation process. A model recently proposed by our groups to describe the poro-viscoelastic behavior of brain tissue (Comellas et al., 2020; Greiner et al., 2021) is here adapted to predict the behavior of the hydrogel. The framework of reference is the Theory of Porous Media, which considers the material as a mixture of an elastic solid phase and an inviscid fluid phase (Ehlers and Eipper, 1999). The long-term equilibrium response of the hydrogel is modeled with a one-term Ogden strain-energy function. Two non-equilibrium branches describe the viscoelastic behavior, each of them with a set of Ogden parameters and a viscosity. Lastly, an isotropic permeability coefficient characterizes the volumetric time-dependent process governed by fluid migration through the network. Using the experimentally derived equilibrium water content of the mimicking material, we estimate the solid volume fraction for the model. The porous structure is verified by imaging with cryo-scanning electron microscopy. To determine all the above parameters, an inverse parameter identification scheme is developed for the proposed indentation experiments. Finally, we validate the model through a finite element simulation of an additional experimental indentation not used for parameter calibration.

## 2 Materials and methods

### 2.1 Hydrogel synthesis and sample preparation

Polyvinyl alcohol granulate (Mw 146,000—186,000, 99+% hydrolized) and Phytagel powder were purchased from Sigma-Aldrich, St. Louis, United States. Deionized water (DIW) was prepared on site by purifying regular tap water with an ion exchanger (TKA DI 2800 S, TKA Wasseraufbereitungssysteme, Niederelbert, Germany). The electrical conductivity was constantly monitored and kept below an upper limit value of 0.1 μS/cm at room temperature.

**FIGURE 1:**
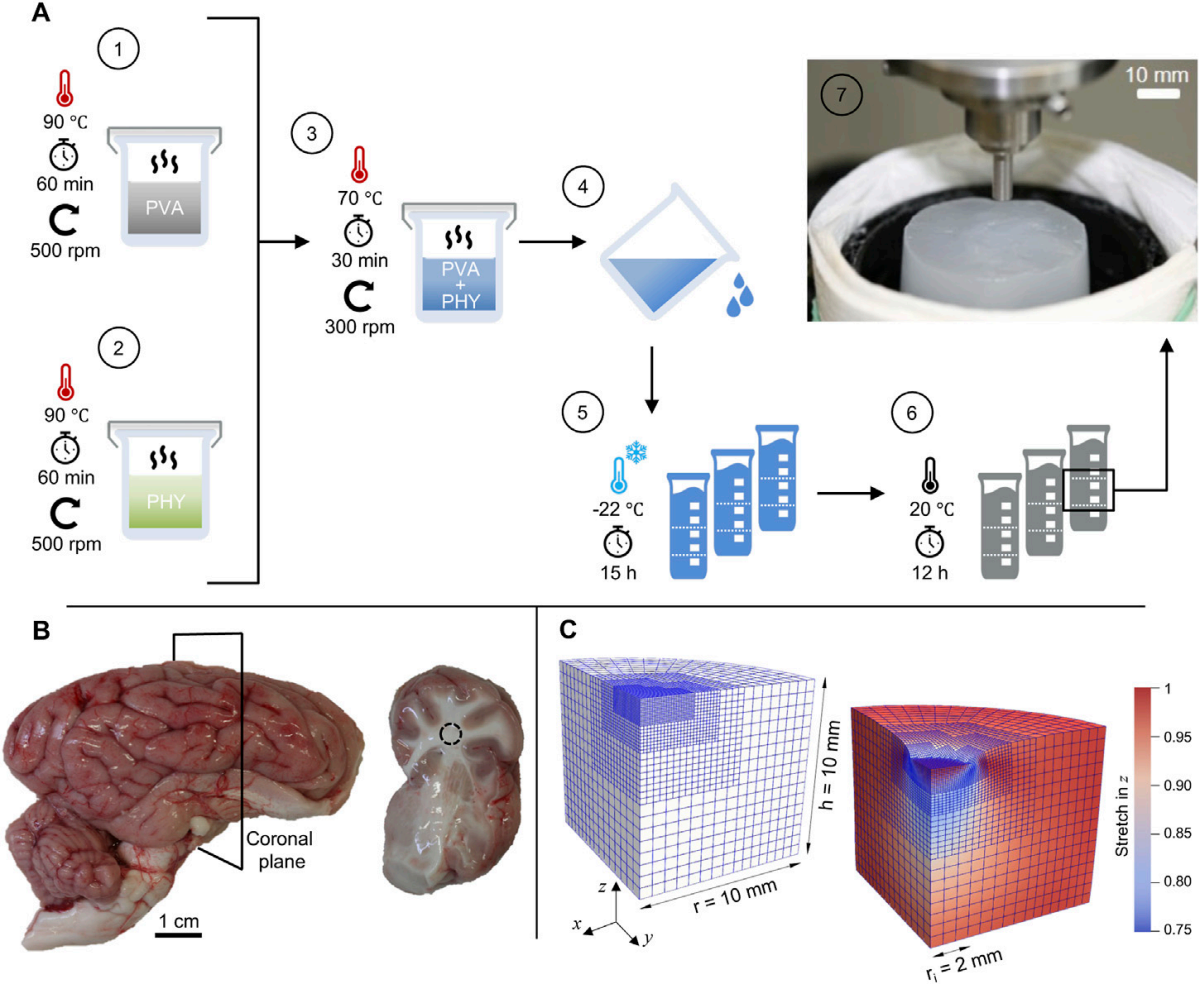
Preparation methods and materials: **(A)** Schematic representation of the standardized preparation procedure for hydrogel synthesis with polyvinyl alcohol (PVA) and Phytagel (PHY). The inset in step 7 shows a hydrogel sample on the sample stage during an indentation test. **(B)** Porcine brain hemisphere with cerebrum, cerebellum and brain stem. The slices (right image) were obtained by cutting along the coronal plane. **(C)** Finite element model in undeformed (left) and deformed configuration (right) for simulating the flat-punch indentation experiment with the poro-viscoelastic model.

Based on the findings of Forte et al. (2016) we have established a standardized preparation procedure for repeatable hydrogel synthesis. Figure 1A schematically shows the workflow with the preparation steps in ascending order. PVA mixtures were obtained by dissolving 6.0 wt% granulate in DIW, heating to 90 °C and agitating on a magnetic stirrer at the target temperature for 60 min (step 1). Phytagel mixtures containing 0.8 wt% powder were prepared following the same procedure (step 2). To obtain a batch of material, we mixed 400 g of each mixture (1:1 weight ratio) and heated the mixture for an additional 30 min at 70 °C (step 3). During the last 15 min we reduced the speed of the magnetic stirrer to a minimum to avoid trapped air bubbles. A casing made from multiple layers of commercially available aluminum foil helped us maintain a stable temperature and retain evaporated DIW in the beaker during synthesis. We divided the liquid compound into plastic molds with a diameter of 40 mm and a volume of 115 mL (step 4). After cooling down for 120 min, the compounds were physically cross-linked by applying a single freeze-thaw cycle (Kim et al., 2015; Tan et al., 2018; Zhao et al., 2022). For this purpose, the samples were frozen at −22 °C for at least 15 h and then thawed for at least 12 h (steps 5 and 6). In the course of this work, the samples were randomly selected from five different material batches (each containing 800 g composite hydrogel) and processed for mechanical and structural characterization. For the indentation tests, we developed a custom-made, 3D-printed cutting mold to produce consistent discs with a diameter of 40 ± 1 mm and a height of 20 ± 1 mm.

For cryo-scanning electron microscopy, we prepared cylindrical samples using a stainless-steel biopsy punch (Stiegelmeyer GmbH and Co. KG) with a nominal (outer) diameter of 8 mm. The samples were trimmed to a height of 8 ± 1 mm using feather trimming blades (Feather Safety Razor CO., Ltd, Japan). Prior to mechanical testing and structural investigations, the hydrogel samples were stored in DIW for 120 min to prevent dehydration and to ensure a fully fluid-saturated material.

### 2.2 Brain sample preparation

We collected fresh porcine brain hemispheres from eight different animals at a local slaughterhouse (Norbert Marcher GmbH, Facility Graz). Until processing for mechanical testing, the samples were stored as a whole in phosphate-buffered saline (PBS) and maintained within a temperature range of 5 °C–10 °C. All samples were processed within a maximum of 6 h post-mortem time (Fountoulakis et al., 2001; Garo et al., 2007; Faber et al., 2022). The brain hemispheres were cut into coronal slices to expose the region around the corona radiata. All sectioned samples were processed immediately after preparation and kept hydrated with PBS to avoid tissue dehydration. Since we chose a maximal indentation depth of 1 mm in the indentation test protocols (see Section 2.3.2), only slices with a thickness of at least 10 mm were considered for the subsequent mechanical tests (van Dommelen et al., 2010; Finan et al., 2014). Figure 1B shows an exemplary sample of a brain hemisphere and a resulting coronal slice.

### 2.3 Mechanical testing

#### 2.3.1 Experimental setup

All mechanical tests described in this work were performed using a triaxial testing device introduced earlier by our groups (Sommer et al., 2013; Budday et al., 2017a). The setup was operated in a displacement-controlled mode with a maximum displacement rate of 100 mm/min along the vertical axis. For the indentation tests described in this work, we modified and updated the original triaxial testing device with a 3D-printed sample stage with a diameter of 70 mm (see Supplementary Material S8). Compared to the initial punch-shaped sample holder with a maximum diameter of 15 mm, the updated stage provides an increased contact area and ensures the collection of the applied hydration fluid during testing. We conducted all indentation tests with a circular flat-punch indenter. The in-house manufactured tool is made of stainless steel with a polished indenting tip of radius *R*_i_ = 2 mm. Unless otherwise noted, all experiments were performed at a temperature of 22 °C ± 3 °C.

#### 2.3.2 Indentation tests

Coronal brain slices and hydrogel discs were characterized *via* indentation with a flat punch (see Figure 1A, inset). To probe the rate-sensitivity of the materials, we used two different indentation protocols, hereinafter referred to as P1 and P2. In test protocol P1, the indenter was moved against the sample surface with a loading speed *v* = 1 mm/s. In protocol P2, the loading speed was *v* = 1.6 mm/s. Because the initial height of each slice/disc varied due the preparation methods, the actual height of the samples was derived individually from contact-force measurements. The indenter tip was brought into contact with a constant speed of 5 mm/min (0.083 mm/s) until a reaction force of 1 mN was recorded. Thereafter, the tip was retracted a distance of −0.1 mm with the same speed until the forces along all three orthogonal axes were equilibrated. The recovery time between the contact force measurement and the first indentation cycle was 60 s (van Dommelen et al., 2010). After this recovery period, the indenter was moved towards the sample surface and held there for 90 s at the target indentation depth of 1 mm. The indenter was then retracted with an unloading speed of 1 mm/s and held at a constant distance above the surface for a period of 60 s. This phase is indicated by the horizontal sections at −3.8 mm (P1, black) and −3 mm (P2, red) in Figure 4A. Before the next indentation cycle, the indenter was again positioned at a distance of 0.1 mm above the sample surface for a period of 60 s. Here, a complete indentation protocol consists of six indentation cycles, each including indentation, holding time of 90 s, unloading and recovery of 120 s. In Figure 4A, a complete cycle is highlighted by the shaded area. Apart from the loading speed and the indenter position during the recovery phase, all other test parameters are identical between the two protocols. The two complete trapezoidal loading profiles (i.e., the time-displacement curves P1 and P2) are illustrated in Figure 4A,B. Because the time-displacement curves are initially synchronized, the two lines deviate over time. Figure 4B shows a magnification of the second loading cycle, with the higher loading speed of protocol P2 (*v* = 1.6 mm/s) clearly indicated by a steeper slope compared to the time-displacement curve of protocol P1 (*v* = 1.0 mm/s).

At the beginning of each indentation test, PBS (for brain) or DIW (for hydrogel) was applied to the surface of the samples to minimize adhesion between the surface and the indenter tip and to avoid potential related tissue damage (Budday et al., 2020a). Each sample was placed on taped sandpaper (P120) to prevent slipping during loading. We ran 10 cyclic indentation tests on each protocol and material, resulting in a total of 240 indentation cycles.

### 2.4 Equilibrium water content

The equilibrium water content of the composite hydrogels was derived using the gravimetric method. Hydrogel discs (*n* = 6) were hydrated in DIW for 120 min after mechanical characterization. To derive the equilibrated wet weight (*m*_wet_), the samples were removed from the DIW, gently blotted to remove excess water on the surfaces, and weighted on a microbalance (Kern ABJ, Kern and Sohn GmbH, Balingen, Germany) in triplicates. The dry state of the samples was obtained by dehydration at ambient temperature over the course of 14 days. Each day, the dry weight (*m*_dry_) of each sample was measured in triplicates. Following the procedure mentioned by (Liu et al., 2015), and with Δ*m*_F_ = *m*_wet_ − *m*_dry_, the water volume fraction was approximated by

$$\phi =\frac{\frac{\Delta m_{\text{F}}}{\rho_{\text{F}}}}{\frac{\Delta m_{\text{F}}}{\rho_{\text{F}}}+\frac{m_{\text{dry}}}{\rho_{\text{dry}}}},$$ (1)

where *ρ*_F_ denotes the mass density of the fluid and *ρ*_dry_ the one of the dry hydrogels, respectively. Here, we used *ρ*_*F*_ = 1.0 g/cm^3^ for the DIW. With the assumption *ρ*_dry_ ≈ *ρ*_F_, *ϕ* can be regarded as the initial fluid volume fraction in the fully-saturated state.

### 2.5 Cryo-scanning electron microscopy

The microstructure of the composite hydrogels was examined with cryogenic scanning electron microscopy (cryo-SEM). The cylindrical samples (see Section 2.1) were frozen under slush liquid nitrogen and transferred under a vacuum transfer device into the preparation chamber for subsequent processing and observation. The cryo preparation chamber was connected directly to the GEMINI Sigma 500 scanning electron microscope (Carl Zeiss Microscopy GmbH, Jena, Germany) and included a nitrogen gas cold stage. The samples were fractured, sublimated, and sputter-coated with palladium. The fractured material was subsequently inserted into the SEM sample chamber where it was kept under vacuum conditions during the whole imaging procedure. The images were taken with a Sigma 500VP FE-SEM with a SEM secondary-electron detector (Zeiss Group, Oberkochen, Germany) operated at an acceleration voltage of 5 kV.

### 2.6 Data processing

From the indentation tests, we obtained data for the vertical reaction forces and the corresponding displacements over time. For subsequent numerical processing and visualization, the raw data sets were time-synchronized using MATLAB (The MathWorks, Inc., Natick, United States). Details are provided in (Supplementary Material S1, Section 5).

### 2.7 Nonlinear poro-viscoelastic model

We applied a nonlinear poro-viscoelastic model based on the Theory of Porous Media introduced by Ehlers and Eipper (1999) and previously presented by our groups in Comellas et al. (2020); Greiner et al. (2021) to model the mechanical behavior of the composite hydrogel. Accordingly, the hydrogel is considered as a biphasic, fully-saturated and compressible medium, consisting of individually incompressible solid and fluid constituents. The viscoelastic solid represents the weakly-bonded polymer network saturated by free-flowing solvent molecules. The overall compressibility of the biphasic material is expressed in terms of changing solid and fluid volume fractions *n* ^S^ and *n* ^F^, respectively, with the saturation condition *n* ^S^ + *n* ^F^ = 1.

#### 2.7.1 Continuum kinematics

**FIGURE 2:**
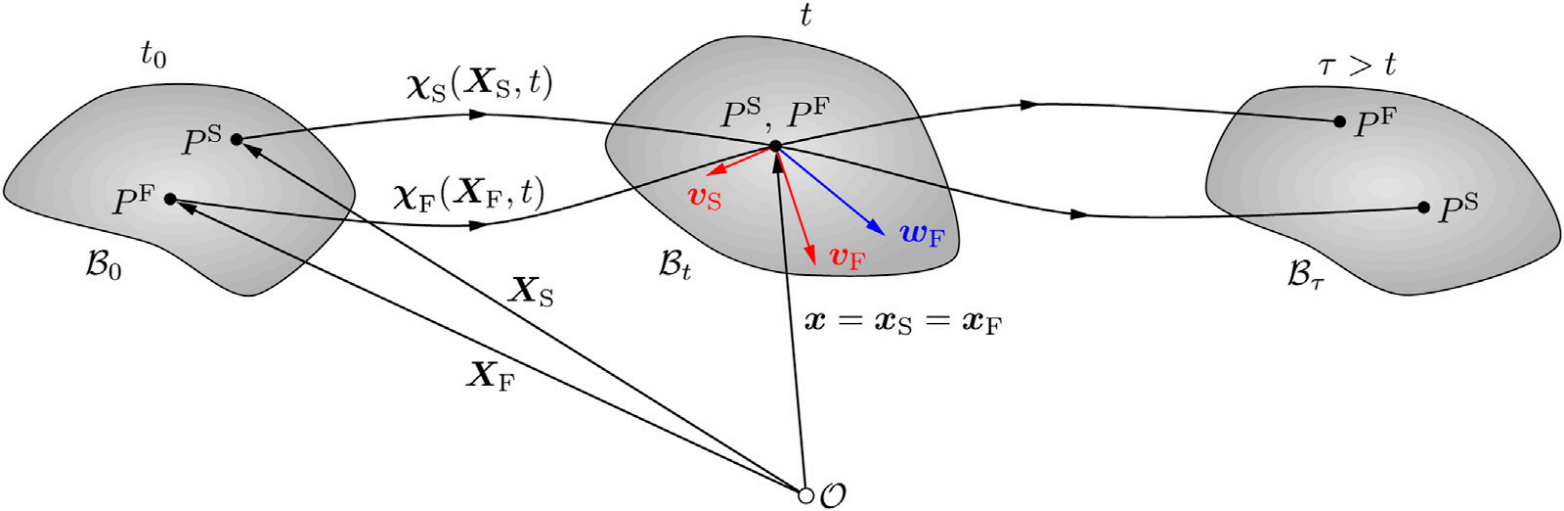
Kinematics of a biphasic material body within the context of the Theory of Porous Media (Ehlers and Eipper, 1999). The material particles of the solid and fluid components (*P* ^S^ and *P* ^F^, respectively) originate from different reference positions in the material configuration $B_{0}$ at the initial time *t*_0_, but occupy the same spatial position in the current configuration $B_{t}$ at time *t*. Adapted from Ehlers (2002).

According to the Theory of Porous Media, the material particles of each constituent proceed from different reference positions ***X***_S_ and ***X***_F_ at time *t*_0_. In the current configuration at a given time *t*, they occupy simultaneously the same spatial position ***x*** (see Figure 2). The corresponding constituent deformation map is $x=\chi_{\text{S}}\left(X_{\text{S}},t\right)=\chi_{\text{F}}\left(X_{\text{F}},t\right)$ . Thus, we obtain the displacement of the solid component

$$u_{\text{S}}=x-X_{\text{S}},$$ (2)

and the material deformation gradient

$$F_{\text{S}}=\frac{\partial x}{\partial X_{\text{S}}}.$$ (3)

In addition, the seepage velocity ***w***_F_ = ***v***_F_ − ***v***_S_ describes the motion of the fluid with respect to the deforming solid, where ***v***_F_ and ***v***_S_ are the velocities of the fluid and solid components, respectively.

#### 2.7.2 Governing equations

We assume quasi-static loading, neglect volumetric forces due to gravity and do not prescribe any external traction vectors. Thereby, the weak form of the linear momentum balance equation in the reference configuration $B_{0}$ reads

$$\int_{B_{0}}\nabla \left(\delta u\right):\tau \;\text{d}V_{0S}=0\;\forall \delta u.$$ (4)

The test function *δ* ***u*** corresponds to the displacement of the viscoelastic solid and d*V*_0S_ refers to the volume element of the biphasic material in the reference configuration of the solid. The constitutive equation of the solid component renders the Kirchhoff stress tensor ***τ***. The mass balance

$$\int_{B_{0}}\delta p\;{\dot{J}}_{\text{S}}\;\text{d}V_{0S}-\int_{B_{0}}\nabla \left(\delta p\right)\cdot wJ_{\text{S}}\;\text{d}V_{0S}=0\;\forall \delta p$$ (5)

introduces the pore pressure test function *δp*, and the constitutive equation of the fluid constituent provides the volume-weighted seepage velocity ***w*** = *n* ^F^ ***w***_F_. The Jacobian *J*_S_ denotes the determinant of the deformation gradient of the solid material *J*_S_ = det ***F***_S_ > 0, and ${\dot{J}}_{\text{S}}$ indicates its material time derivative computed with respect to the motion of the solid component. Note that forced fluid flow across the boundary is not prescribed and that the time dependencies of the mass balance Eq 5 result in a nonstationary nature of the governing equations.

#### 2.7.3 Constitutive equations

We perform a multiplicative split of the solid deformation gradient ***F***_S_ into an elastic part (labeled by superscript e) and a viscous part (labeled by superscript v), i.e.,

$$F_{\text{S}}=F_{\text{S},i}^{\text{e}}\cdot F_{\text{S},i}^{\text{v}}$$ (6)

for *i* = 1, 2 viscoelastic elements arranged in parallel. The addition of a second viscoelastic element to the original poro-viscoelastic formulation proposed by Comellas et al. (2020) was motivated by our experimental findings.

The Kirchhoff stress tensor is expressed as the sum of a term related to the pore pressure *p* and an ‘extra’ part $\tau_{\text{E}}^{\text{S}}$ , which following Eq. 6 is additively split into equilibrium (eq) and non-equilibrium (neq) parts, such that the constitutive equation of the solid is

$$\tau =\tau_{\text{E}}^{\text{S}}-pJ_{\text{S}}1=\tau_{\text{E}}^{\text{eq}}+\overset{2}{\underset{i=1}{\sum }}\tau_{\text{E},i}^{\text{neq}}-pJ_{\text{S}}1,$$ (7)

and **1** denotes the second-order unit tensor. According to previous studies (Budday et al., 2017b; Budday et al., 2020a), a one-term Ogden model is most suitable to capture the complex behavior of brain tissue, including compression-tension asymmetry, conditioning and hysteresis. In addition, the equilibrium term includes a volumetric contribution $\tau_{\text{E}}^{\text{vol}}$ in order to account for the compressibility effects of the deforming biphasic material. Then,

$$\begin{array}{cc} \tau_{\text{E}}^{\text{eq}} & =\overset{3}{\underset{a=1}{\sum }}\beta_{\infty ,a}\;n_{\text{S},a}⊗n_{\text{S},a}+\tau_{\text{E}}^{\text{vol}},\text{with}\\ \beta_{\infty ,a} & =\mu_{\infty }\left[{{\tilde{\lambda }}_{\text{S},a}}^{\alpha_{\infty }}-\frac{1}{3}\left({{\tilde{\lambda }}_{\text{S},1}}^{\alpha_{\infty }}+{{\tilde{\lambda }}_{\text{S},2}}^{\alpha_{\infty }}+{{\tilde{\lambda }}_{\text{S},3}}^{\alpha_{\infty }}\right)\right], \end{array}$$ (8)

where *μ*_*∞*_ and *α*_*∞*_ are the equilibrium Ogden shear moduli and nonlinearity parameters, ***n***_S,*a*_ are the eigenvectors of the left Cauchy-Green tensor $b_{\text{S}}=F_{\text{S}}\cdot F_{\text{S}}^{\text{T}}=\sum_{a=1}^{3}\lambda_{\text{S},a}^{2}\;n_{\text{S},a}⊗n_{\text{S},a}$ , and ${\tilde{\lambda }}_{\text{S},a}=J_{\text{S}}^{-1/3}\lambda_{\text{S},a}$ , *a* ∈ 1, 2, 3, are the isochoric principal stretches (Holzapfel, 2000). Similarly, the non-equilibrium part of the Kirchhoff stress tensor reads

$$\begin{array}{cc} \tau_{\text{E},i}^{\text{neq}} & =\overset{3}{\underset{a=1}{\sum }}\beta_{i,a}\;n_{\text{S},i\;a}^{\text{e}}⊗n_{\text{S},i\;a}^{\text{e}},\text{with}\\ \beta_{i,a} & =\mu_{i}\left[{\tilde{\lambda }}_{\text{S},i\;a}^{\text{e}\alpha_{i}}-\frac{1}{3}\left({\tilde{\lambda }}_{\text{S},i\;1}^{\text{e}\alpha_{i}}+{\tilde{\lambda }}_{\text{S},i\;2}^{\text{e}\alpha_{i}}+{\tilde{\lambda }}_{\text{S},i\;3}^{\text{e}\alpha_{i}}\right)\right], \end{array}$$ (9)

where *μ*_*i*_ and *α*_*i*_ the corresponding Ogden non-equilibrium shear moduli and nonlinearity parameters, $n_{\text{S},i\;a}^{\text{e}}$ are the eigenvectors of the elastic part of the left Cauchy-Green tensor $b_{\text{S},i}^{\text{e}}=F_{\text{S},i}^{\text{e}}\cdot {\left(F_{\text{S},i}^{\text{e}}\right)}^{\text{T}}=\sum_{a=1}^{3}{\lambda_{\text{S},i\;a}^{\text{e}}}^{{\;}^{2}}\;n_{\text{S},i\;a}^{\text{e}}⊗n_{\text{S},i\;a}^{\text{e}}$ , and ${\tilde{\lambda }}_{\text{S},i\;a}^{\text{e}}={J_{\text{S}}^{\text{e}}}^{{\;}^{-1/3}}\lambda_{\text{S},i\;a}^{\text{e}}$ , *a* ∈ 1, 2, 3, denote the isochoric elastic principal stretches. Finally, the volumetric contribution to the solid stress tensor is

$$\tau_{\text{E}}^{\text{vol}}=\lambda^{*}{\left(1-n_{0S}^{\text{S}}\right)}^{2}\left(\frac{J_{\text{S}}}{1-n_{0S}^{\text{S}}}-\frac{J_{\text{S}}}{J_{\text{S}}-n_{0S}^{\text{S}}}\right)1,$$ (10)

where λ* is the first Lamé parameter of the solid constituent, $n_{0S}^{\text{S}}$ denotes the initial solid volume fraction with respect to the solid reference configuration. The volumetric part of the Kirchhoff stress tensor ensures the quasi-incompressibility of the solid since it increases hyperbolically once the deformation reaches the compaction point, i.e., when all pores are closed and no fluid remains in the material (Ehlers and Eipper, 1999).

A Darcy-like law describes the constitutive behavior of the fluid component and provides the volume-weighted seepage velocity

$$w=-\frac{1}{\mu^{\text{FR}}}\frac{J_{\text{S}}-n_{0S}^{\text{S}}}{1-n_{0S}^{\text{S}}}K_{0}^{\text{S}}\cdot \nabla p,$$ (11)

where $n_{0S}^{\text{S}}$ is the solid volume fraction referring to the solid reference configuration. Therein, *μ* ^FR^ is the effective shear viscosity of the pore fluid and $K_{0}^{\text{S}}=K_{0}1$ is the isotropic initial intrinsic permeability tensor. As for the solid component, we neglect gravitational effects on the pore fluid.

To ensure thermodynamical consistency, we derive appropriate dissipation terms for the viscous solid behavior and the porous dissipation from the Clausius-Duhem inequality. We assume isotropy and choose an evolution equation for the internal variables (Comellas et al., 2020)

$$-L_{v_{\text{S}}}b_{\text{S},i}^{\text{e}}\cdot {\left(b_{\text{S},i}^{\text{e}}\right)}^{-1}=\frac{1}{\eta_{i}}\tau_{\text{E},i}^{\text{neq}},$$ (12)

where the symbol $L_{v_{\text{S}}}$ denotes the Lie derivative of the left Cauchy-Green tensor along the velocity field of the solid motion, and *η*_*i*_ are the solid viscosities. The differential Eq 12 *a priori* satisfies a non-negative viscous dissipation density rate for each individual mode *i*, i.e.,

$$D_{\text{v}}=\overset{2}{\underset{i=1}{\sum }}\frac{1}{2\eta_{i}}\tau_{\text{E},i}^{\text{neq}}:\tau_{\text{E},i}^{\text{neq}}\geq 0\text{for}\eta_{i}>0.$$ (13)

The porous dissipation density rate represents the dissipation due to the seepage process related to the material porosity. Given that *μ* ^FR^ and *K*_0_ are necessarily positive and $n_{0S}^{\text{S}}\in \left(0,1\right)$ , the porous dissipation

$$D_{\text{p}}=\frac{\mu^{\text{FR}}}{K_{0}}\frac{1-n_{0S}^{\text{S}}}{J_{\text{S}}-n_{0S}^{\text{S}}}w\cdot w\geq 0$$ (14)

will always be non-negative.

### 2.8 Numerical setup

The open source C++ library deal.ii (Arndt et al., 2020) provides the numerical framework to reconstruct the experimental flat-punch indentation (see Section 2.3) in the context of the Finite Element (FE) method to investigate the behavior of the poro-viscoelastic model. Figure 1C depicts the FE mesh in undeformed and deformed configuration, shows its dimensions and displays the local vertical stretch distribution in the deformed state.

The spatial discretization consists of 3184 full integration Q2P1 elements, i.e., quadratic shape functions for the displacement and linear ones for the pore pressure approximation, resulting in a total of 93808 degrees of freedom. Exploiting symmetry and local mesh refinement with hanging nodes allowed us to accurately resolve the flat-punch indenter tip with moderate computational cost. After some preliminary studies did not show any significant deviations, we decided to reduce the dimensions of the meshed geometry by 50% compared to the real samples. In this way we can further reduce the computational effort for each simulation, which contributes to an important speedup of the inverse parameter identification scheme (see Section 2.9).

The bottom of the geometry is fully fixed in space and a vertical displacement is applied to all degrees of freedom within the radius of the flat punch while their in-plane position remains fixed, i.e., full adhesion is assumed. We import the corresponding time-displacement curve directly from the experimental output to avoid any discrepancies with respect to the actual loading protocol. Symmetry boundary conditions apply to the inner lateral surfaces. Except for the loaded part of the top surface, all of the outer surfaces are drained such that fluid can leave (or enter) the solid through the boundary.

Table 1 lists all material parameters. The value of the initial solid volume fraction $n_{0S}^{\text{S}}$ is derived from the experimentally determined equilibrium water content in Section 3.1.2 under the assumption that the fluid is completely free-flowing through the solid matrix. To ensure the solid quasi-incompressibility, we set the first Lamé parameter λ* to a reasonably high value. The effective shear viscosity *μ* ^FR^ of the pore fluid is that of water at room temperature. All other material parameters, i.e., the equilibrium and non-equilibrium shear moduli, Ogden nonlinearity parameters, solid viscosities and the initial intrinsic permeability are derived from an inverse parameter identification scheme described below.

**TABLE 1:** Poro-viscoelastic material parameters for the composite hydrogel. The initial solid volume fraction $n_{0\text{S}}=1-\setminus \varphi$ was estimated experimentally (see Figure 5 and Section 2.4), while the first Lamé parameter λ* was set to a reasonably high value that ensured solid quasi-incompressibility. The effective shear viscosity *μ* ^FR^ corresponds to that of water at room temperature. Our inverse parameter identification (see Section 2.9) varies the parameters in given intervals and finally provides an optimal set of all remaining parameters.

Parameter	Interval	Value	Unit
Solid component (fixed parameters)			
$n_{0S}^{\text{S}}$		0.032	
λ*		1.00 ⋅ 10^5^	Pa
Solid component (fitted parameters)			
*μ*_*∞*_	[−1000, − 1]	−217	Pa
*μ*_1_	[−1000, − 1]	−418	Pa
*μ*_2_	[−1000, − 1]	−161	Pa
*α*_*∞*_	[−30, − 1]	−2.16	
*α*_1_	[−30, − 1]	−13.5	
*α*_2_	[−30, − 1]	−2.97	
*η*_1_	[1, 100000]	50.6	Pa⋅s
*η*_2_	[1, 100000]	4.68 ⋅ 10^3^	Pa⋅s
Fluid component (fixed parameters)			
*μ* ^FR^		0.89 ⋅ 10^–3^	Pa⋅s
Fluid component (fitted parameters)			
*K*_0_	[10^–14^, 10^–2^]	2.30 ⋅ 10^–9^	mm^2^

### 2.9 Inverse parameter identification scheme

**FIGURE 3:**
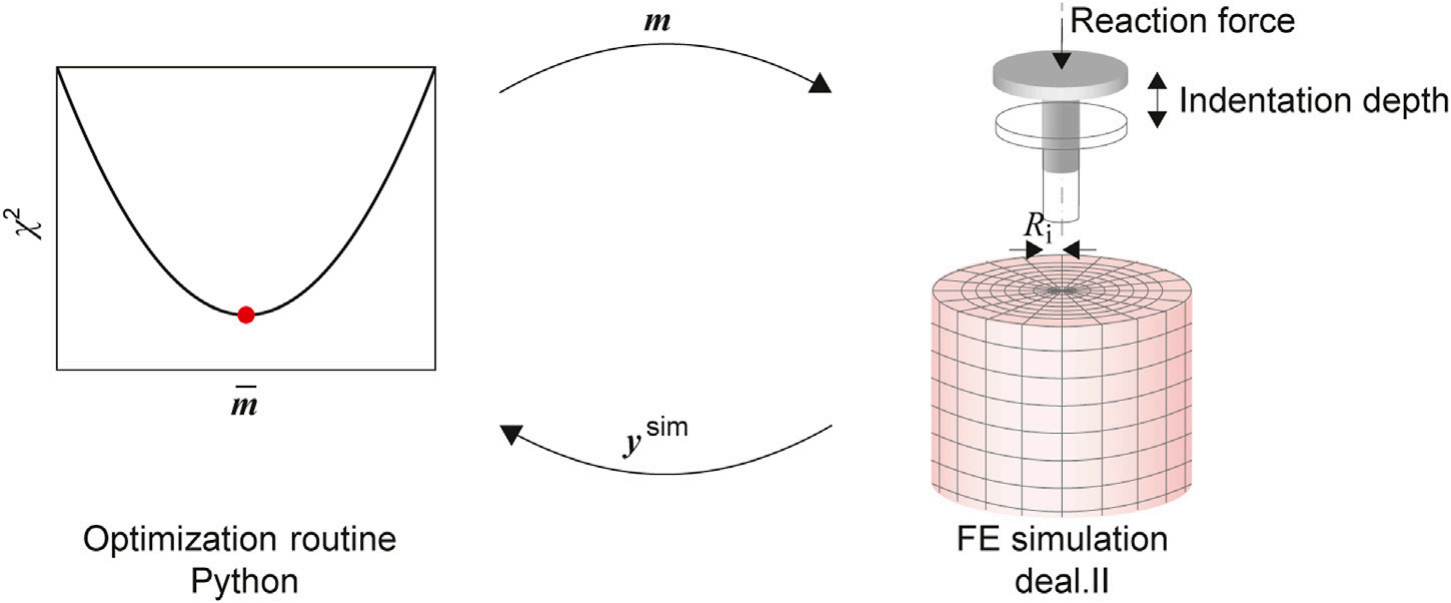
The parameter identification consists of an optimization routine, implemented in the Python module SciPy, coupled with the FE model solved using deal.ii. The material parameters in the input vector ***m*** are updated to minimize the L2 Norm *χ* ^2^ of the residual vector, calculated from the experimental output and the output of the simulation ***y*** ^sim^.

An inverse parameter identification scheme (Hinrichsen et al., 2022) is used to find the best fitting material parameters ***m*** for a model G(m) so that the model output reproduces the experimental results ***d***. This relation can be written as

$$G\left(m\right)=d,$$ (15)

where G is the FE model used to simulate the conducted experiments and the output is expressed in terms of the resulting reaction forces. The task of identifying the optimal set of material parameters $\bar{m}$ that minimizes the squared error *χ* ^2^ between experimental and simulated values is an inverse problem. By denoting the experimental response values as ***y*** ^exp^ and the simulated values as ***y*** ^sim^, we can define the error function as

$$\chi^{2}=\frac{\sum_{i=1}^{N}{\left(y_{i}^{\exp}-y_{i}^{\text{sim}}\right)}^{2}}{\sum_{i=1}^{N}{\left(y_{i}^{\exp}\right)}^{2}},$$ (16)

where the total number of measured and simulated values is described by *N*. The scaling factor $\sum_{i=1}^{N}{\left(y_{i}^{\exp}\right)}^{2}$ was proposed by Gavrus et al. (1996) and is introduced to deal with the problem of vanishing gradients of *χ* ^2^ for small numerical values. For the minimization of *χ* ^2^ we use the trust-region-reflective algorithm, suited to solve bounded nonlinear least-square problems (Branch et al., 1999). We use a modified version of the implementation found in the Python package SciPy (Virtanen et al., 2020). Figure 3 shows a sketch of the inverse optimization scheme.

As the FE model leads to computationally expensive evaluations of the cost function, some modifications have been implemented to improve the overall efficiency. This is achieved by parallelizing the simulation runs required for the finite difference approximation of the Jacobian. Also, we switched to a so-called eager evaluation, where a new function evaluation—corresponding to a run of the FE simulation—is started as soon as the optimization algorithm calculates a new vector of material parameters for which such an evaluation is required. By implementing a cache of simulation outputs, we can prevent unnecessary simulation runs by comparing the already processed cached parameters with those requested by the optimization algorithm. Finally, based on preliminary studies, initial values for the material parameters and intervals (see Table 1) are prescribed.

## 3 Results

### 3.1 Experimental results

The experimental part of the work included the preparation and adaptation of the composite material together with the associated mechanical tests. We established a benchmark by performing indentation experiments on porcine brain tissue for direct comparison. We dehydrated the hydrogels to experimentally determine the water content and estimate the solid volume fraction for subsequent modeling. With cryo-scanning electron microscopy, we imaged the internal structure of the synthetic material.

#### 3.1.1 Mechanical testing

**FIGURE 4:**
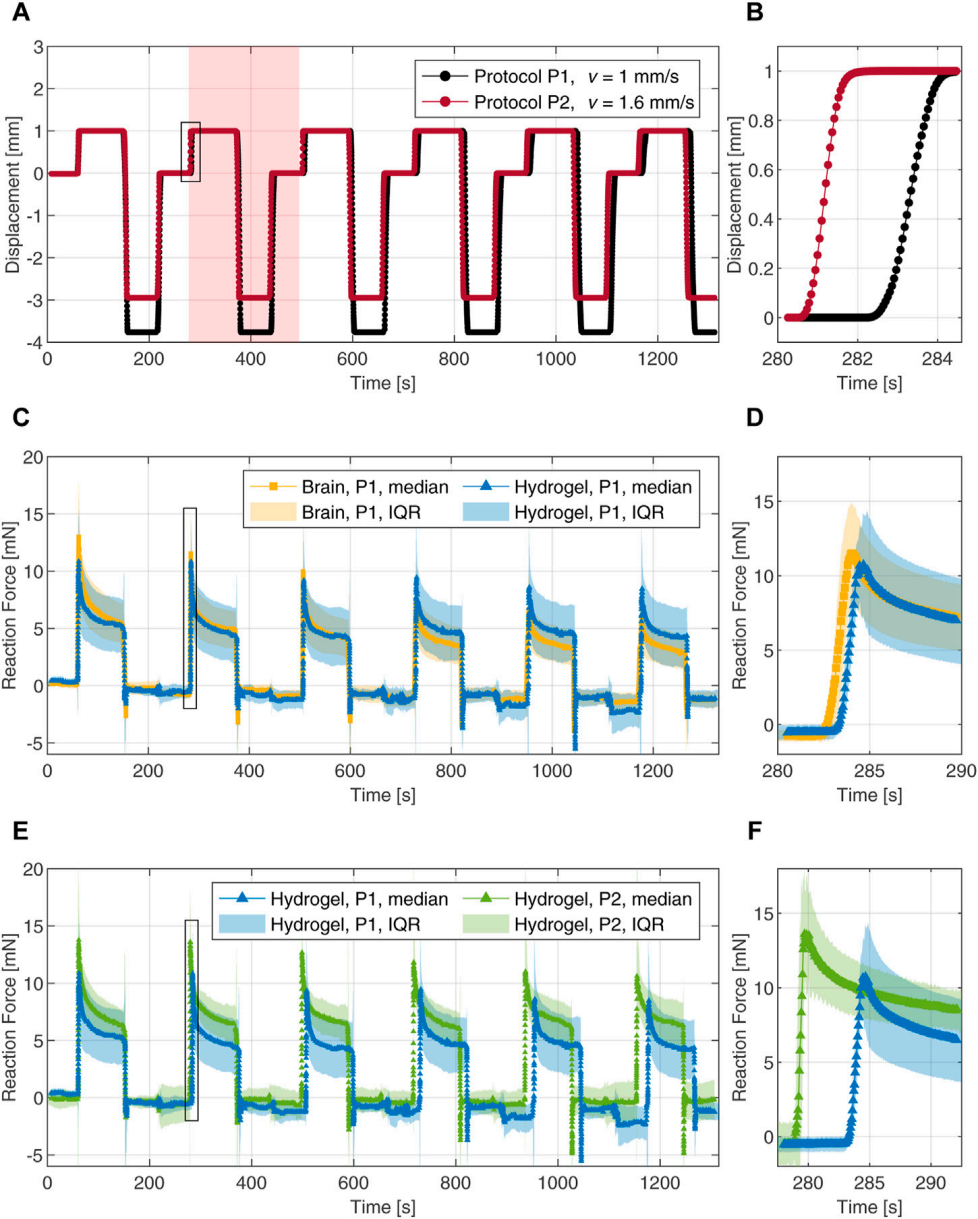
Cyclic indentation tests with a 4 mm flat-punch. **(A)** Loading protocols with two different speeds represented by time-displacement curves. The shaded area highlights one complete indentation cycle. **(B)** Magnification of the loading part during the second indentation cycle. **(C)** Force-time responses of the composite hydrogel (blue) and porcine brain tissue (yellow) during six indentation cycles. **(D)** Magnification of the force-time response upon loading during the second cycle. **(E)** Force-time responses of the composite hydrogel probed with two different loading rates. **(F)** Magnification of the force-time response upon loading during the second cycle. The data is represented by the median and the interquartile range (IQR). The magnifications in the right column are marked with black frames in the corresponding figures to the left.

First, we compared the mechanical responses of the hydrogel and brain tissue *via* flat-punch indentation. The trapezoidal time-displacement curves for the two different loading speeds, shown in Figure 4A, B, are described in Section 2.3.2.

To assess the ability of the tuned composite hydrogel to mimic brain tissue behavior, we compared the mechanical response of both materials over all six indentation cycles. The median data, derived from 10 individual force-time curves in each case, were used as a measure of the central tendency of the experimental data. The dispersion of the data was derived by computing the interquartile range (IQR) for each time step. The IQR represents the spread of the middle 50% of the data around the median. The reaction forces over time for protocol P1 are shown in Figures 4C, D. Both materials exhibit a nearly linear response at initial loading with median peak forces of 11 mN and 13 mN for the hydrogel and the brain tissue, respectively; Figure 4D shows a magnification of the second loading cycle with a median peak force of 11 mN for the hydrogel and a value of 12 mN for the brain tissue. The force relaxation (i.e., the difference between the peak force and the relaxed force divided by the peak force) after the first indentation cycle is 52% for the hydrogel and 58% for the brain tissue. Force relaxation after the second cycle increased to 60% for the hydrogel while staying at 58% for the brain tissue. After the third indentation cycle, the measured forces on the brain tissue decrease gradually and faster compared to the hydrogel. In the final indentation cycle, we observed a median peak force of 9 mN for the hydrogel and 6 mN for the brain tissue measurements. Force relaxation after six indentation cycles was 50% for both materials.

The rate-dependent behavior was investigated by comparing both indentation protocols on the hydrogel. The reaction forces (median with interquartile range) *versus* time are shown in Figures 4E, F. The material exhibits significantly different responses when tested at the two different loading speeds. The responses upon loading during the second cycle are highlighted in Figure 4F. Compared to the median peak forces observed with protocol P1, the peaks obtained with protocol P2 are increased by 27% in cycle 1, 27% in cycle 2 and 24% in indentation cycle 6. The force relaxations after 90 s of indentation arrived at 55% and 53% after the first and the second cycles, respectively. In the sixth indentation cycle with the faster loading, the force decreased by 40% after 90 s.

#### 3.1.2 Equilibrium water content and structural features

**FIGURE 5:**
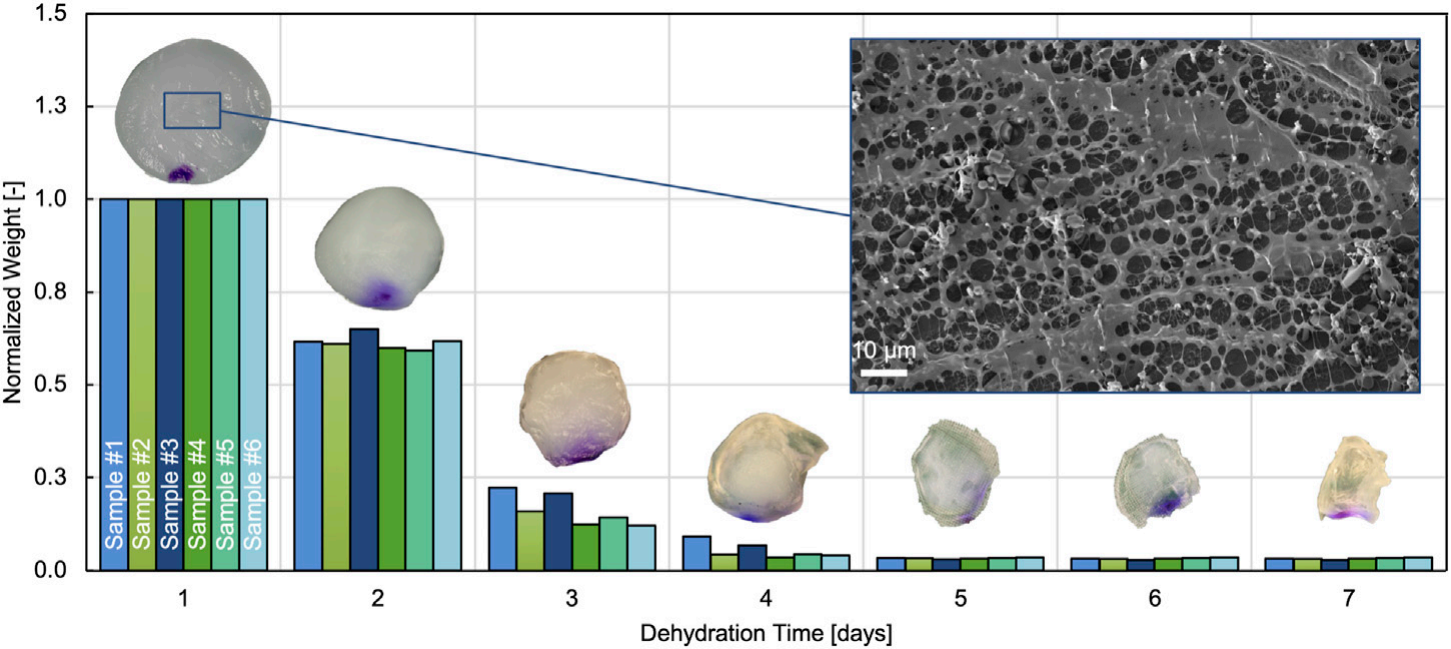
Evolution of normalized weight over dehydration time for 6 different samples. Only the first 7 days are shown because no weight changes could be measured after that. The inset on top of each bar group illustrates representative hydrogel samples on the respective day of dehydration. Also included is a representative SEM image of the hydrogel microstructure.

We tracked the weight of the hydrogel samples over a 14 day period of dehydration. Figure 5 shows the weight evolution during the first 7 days, with each of the measured values normalized by the corresponding equilibrated wet weight (*m*_wet_), measured on the first day. The equilibrium water content was derived by evaluating Eq. 1 using the dry weight (*m*_dry_) measured after 14 days together with the corresponding wet weight (*m*_wet_) of each sample. Assuming that all fluids are free-flowing water and based on the approximations given in Section 2.4, we derived the equilibrium water volume fraction with $\phi \equiv n_{0,\exp}^{\text{F}}=0.968\pm 0.002$ (mean ± standard deviation). Since we treat the hydrogel as a biphasic material with the saturation condition *n* ^S^ + *n* ^F^ = 1, we assessed the solid volume fraction in the fully-saturated state as $n_{0S,\exp}^{\text{S}}\approx 0.032$ .

The microstructure of the hydrogel was imaged using cryo-SEM. A representative image is shown in Figure 5. The brighter details in the foreground represent the exposed porous hydrogel network on the surface after sublimation. The dark background shows the hydrogel with the water still enclosed in the composite.

### 3.2 Computational results

We used the inverse parameter identification scheme described in Section 2.9 to fit the first indentation cycle of the composite hydrogel tested with loading protocol P2. With reasonable initial values for each parameter, the optimization algorithm found an optimal set of material parameters within 20 iterations. Table 1 depicts the resulting material parameters for the solid and fluid components and shows that all parameters are well within the prescribed parameter intervals.

From the equilibrium parameters *μ*_*∞*_ and *α*_*∞*_ of the viscoelastic Ogden model introduced in Section 2.7, we can derive the equilibrium shear modulus $\mu_{\infty }^{0}=\frac{1}{2}\mu_{\infty }\alpha_{\infty }=234$ Pa, and, similarly, for the non-equilibrium parts $\mu_{1}^{0}=2.82$ kPa and $\mu_{2}^{0}=239$ Pa. From the solid viscosities *η*_1_ and *η*_2_, we derive the associated relaxation times describing the short and long-term relaxation behavior $\tau_{i}=\eta_{i}/\mu_{i}^{0}$ of *τ*_1_ = 0.018 s and *τ*_2_ = 19.6 s. The initial intrinsic permeability results in *K*_0_ = 2.3 ⋅ 10^–9^ mm^2^.

**FIGURE 6:**
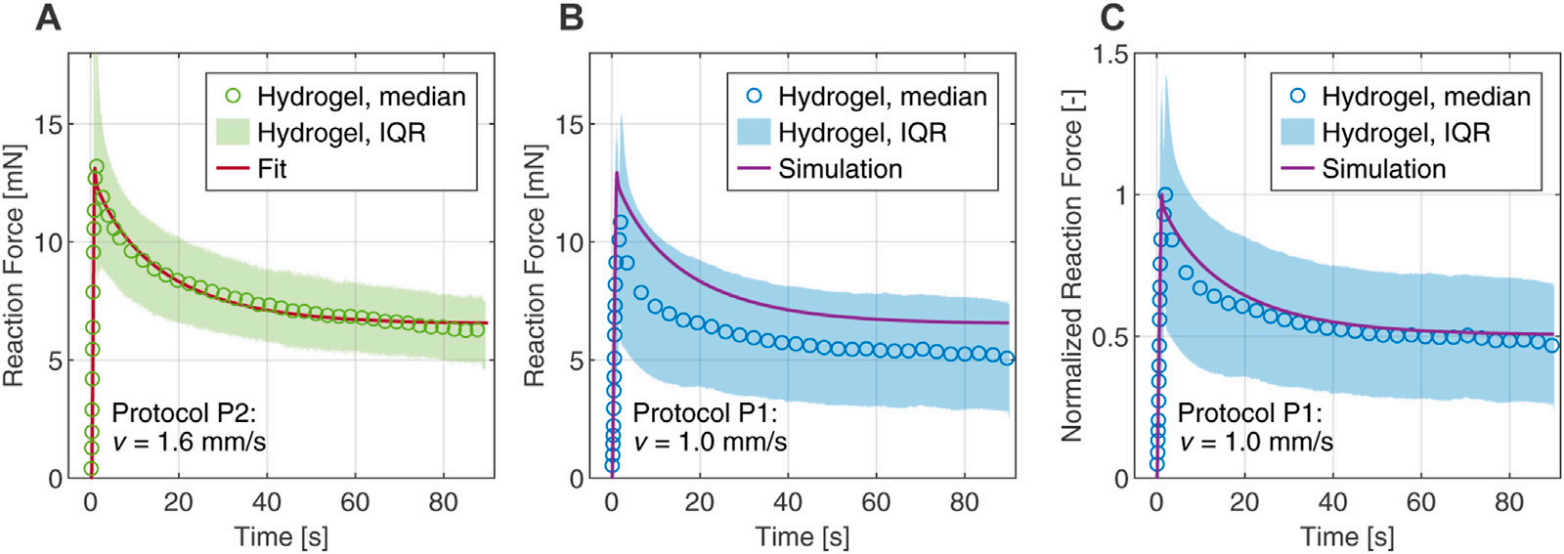
Comparison between computational and experimental results in the first indentation cycle of the composite hydrogel. The experimental data are represented by the median and interquartile range (IQR). **(A)** Fitted reaction force of protocol P2 obtained from the inverse parameter identification scheme. **(B)** Numerical simulation of protocol P1 with material parameters determined from the fit. **(C)** Reaction force of protocol P1 normalized by the peak forces, comparing experiment and simulation.

Figure 6A compares the reaction forces from experimental data with the numerical fit and shows excellent agreement in peak force and relaxation behavior for protocol P2. As a validation of the calibrated poro-viscoelastic model, we used the same material parameters to simulate the slower loading protocol P1. Figure 6B shows some offset in the predicted reaction force compared to experiments, but it is still in the interquartile range. In addition, Figure 6C shows good agreement for the reaction force normalized by the peak force, indicating that the relaxation behavior is captured satisfactorily by the model.

## 4 Discussion

In this work, we prepared a composite hydrogel and compared its mechanical response during cyclic indentation to that of porcine brain tissue. For the first time, the constitutive parameters of the mimicking material were derived directly by fitting experimental data to the introduced poro-viscoelastic material model through an inverse parameter identification scheme. To determine the solid volume fraction of the investigated material, we measured the equilibrium water content. Finally, we validated the obtained material parameters by finite element simulations of additional experiments with different loading rates.

### 4.1 Hydrogel preparation and indentation testing

The hydrogel used here, which owes its characteristic properties to the ratio of polyvinyl alcohol (PVA) and Phytagel (PHY), was introduced by Forte et al. (2016). Based on their findings, we refined the preparation protocol for repeatable material synthesis. Maintaining a stable temperature while mixing the individual solutions was critical. A casing made of several layers of aluminum foil helped us to keep the temperature stable. It also helped prevent the evaporated water from escaping from the beakers during material synthesis. With this modification, we found that solutions containing 6 wt% PVA and 0.8 wt% PHY, respectively, resulted in a composite material best matched the properties of brain tissue.

We evaluated the mimicking nature of the hydrogel through a qualitative comparison of the complete set of force-time responses, including all cycles of the indentation protocol. In fact, the complex nature of brain matter cannot be reduced to a comparison based on a single quantitative parameter such as material stiffness (Axpe et al., 2020). Our approach is able to cover a wide spectrum of material responses, including viscoelasticity with its characteristic relaxation, adhesive behavior and structural features resulting from the porous nature of this tissue (Bilston, 2011; Budday et al., 2020b).

Figure 4 shows the synchronized and averaged data for the entire indentation experiments. At the end of each indentation cycle, the force reaches a distinct negative peak, after which it approaches zero again. Those peaks are due to adhesive effects and occurred when the indenter was removed from the surface. The liquid film initially adheres to the indenter tip and prevents the surface from dehydration. The (negative) force increased until the liquid film between the indenter tip and the surface broke. A possible explanation for this was reported elsewhere (Suh et al., 1995). When the fluid-saturated porous hydrogel expands after being compressed during the indentation process, the pore fluid is depleted to some extent. A negative pore pressure then creates a suction effect on the indenter tip. The macroscopic consequence is expressed as adhesion of the indenter to the porous sample surface. We observed this adhesive behavior for both materials, the coronal sections of porcine brain tissue and the hydrogel discs. In some tests, traces of the liquid remained on the indenter, causing deviations from the zero line in the force-time diagram between indentations. We only applied the liquid to the surface at the beginning of each measurement, since any additional manipulation during the measurement would have affected the recorded forces.

Our results in Figures 4C, D confirm a close agreement between the time-dependent behavior of the hydrogel and native brain tissue. From the third cycle there is only a slight deviation of the median curves. The overlap of the individual mechanical responses together with the agreement of the median peak forces and the median force relaxation values (see Section 3.1.1), indicate that the material indeed exhibits tissue-mimicking behavior.

The indentation protocol used here reliably captured the mechanical response of the coronal slices. However, the results represent the *ex vivo* condition, which may not be reliable to predict the mechanical properties of *in situ* brain tissue (Gefen and Margulies, 2004; Guertler et al., 2018; Budday et al., 2020a). It should therefore be noted that the material in our study closely mimic the *ex vivo* behavior of brain tissue, particularly under indentation loading. Nevertheless, earlier studies suggest that the *in vivo* behavior can also be captured with the composite material (Gefen and Margulies, 2004; Forte et al., 2016).

In this work, we describe the constitutive behavior without considering damage or fracture. Considering these additional mechanisms is crucial for the simulation of surgical procedures, where fractures occur through the interaction of surgical tools with the tissue (Terzano et al., 2021). Future research will focus on extending the behavior of materials that mimic brain tissue in the indentation tests presented here.

### 4.2 Equilibrium water content

We derived the water content of the hydrogel in a fully-saturated state by dehydration. The approximation of the solid volume fraction based on the gravimetric method (i.e., solid mass fraction) is a commonly used technique to characterize polymeric hydrogels (Liu et al., 2015; Richbourg et al., 2022). We followed the approach of Liu et al. (2015) and assumed the density of the dry hydrogel is the same as that of the pore fluid. This assumption holds when the volume reduction of the hydrogel samples equals the mass reduction. If this were not the case, testing the mass density of the hydrogel in the dry state would lead to a more accurate estimation of the equilibrium water content.

We note that the dehydration method did not allow us to determine how much water can be considered as free-flowing fluid. So we assumed that all the water could move freely. Studies on similar hydrated polymer systems have shown that the water exists in different states (Sekine and Ikeda-Fukazawa, 2009; Kudo et al., 2014). Depending on the state of the water (free-flowing, bound or an intermediate state), the contribution to the mechanical response varies. The state of the water within the composite hydrogel has not been investigated in detail. However, when investigated with cryo-SEM, the hydrogel network shows a regular arrangement of pores in which fluid can flow (see Figure 5). In the future, a systematic characterization of the material’s microstructure and quantification of the actual free-flowing water could improve the model predictions.

### 4.3 Parameter identification and model performance

For modeling the highly nonlinear, biphasic material behavior under complex deformations, e.g., in flat-punch indentation tests, we applied our poro-viscoelastic formulation (Comellas et al., 2020), which was developed within the framework of nonlinear continuum mechanics and the Theory of Porous Media. In order to fully capture the relaxation behavior of the hydrogel in the indentation experiments, a second viscoelastic element was added to the original formulation. Then we combined the finite element simulations with an inverse parameter identification scheme (Hinrichsen et al., 2022). The resulting optimal set of material parameters gives us an excellent fit of the experimental data under loading protocol P2 (see Figure 6A). Furthermore, the simulation of the indentation protocol P1 is well within the scatter of the experimental data, which is represented here by the interquartile range (see Figure 6B). The corresponding relaxation behavior is captured almost perfectly (see Figure 6C) and serves as a validation of the predictive ability of the model. In the future we want to extend the numerical investigations to explore the applicability of this parameter set and its possible variations in different load cases.

However, there is a slight mismatch between the force relaxation curves in Figure 6B, indicating an overall stiffer response in the simulation. We attribute these deviations to the restricted relaxation time of 90 s of a single indentation cycle, which was taken from the literature (Forte et al., 2016) and has contributed advantageously to reduce the computation time required during the fitting procedure. Figure 4E shows that the forces are not balanced after 90 s as they reach different values for the loading speeds in P1 and P2. This indicates that the timescales chosen in the indentation protocols are not sufficient for the hydrogel to reach a fully relaxed state, which is only reached after approximately 400 s–600 s (see; Supplementary Material S8). From a computational point of view, this means that the inverse parameter identification scheme delivers unsuitable values for the equilibrium parameters of the model, i.e., values that are too high. This, in turn, also slightly affects the non-equilibrium parameters, resulting in an overall stiffer response.

The parameter identification scheme provides the initial intrinsic permeability with *K*_0_ = 2.3 ⋅ 10^–9^ mm^2^. To the best of our knowledge, this is the first time that the intrinsic permeability has been derived directly from fitting experimental data for this brain tissue-mimicking material. The poro-viscoelastic model previously proposed for the composite PVA-Phytagel hydrogel (Forte et al., 2018) employed a hydraulic conductivity derived from literature data of human brain tissue (Kaczmarek, 1997). The intrinsic permeability is related to the hydraulic conductivity *k* *via* *k* = *K*_0_ *ρ*_F_ *g*/*μ* ^FR^. Using this and the gravitational constant *g* = 9.81 m/s^2^ we derive the hydraulic conductivity for the composite hydrogel to be *k* = 2.5 ⋅ 10^–8^ m/s. This value is well within the proposed range (Forte et al., 2018). Recently published data reporting direct measurements of the hydraulic permeability of brain tissue show a similar magnitude (Vidotto et al., 2019; Jamal et al., 2021). Nevertheless, our derived intrinsic permeability must be treated with caution, as further investigations focusing on the pressure-flow dependence of permeability are required. A variation of the numerical values of the permeability and the sensitivity of the material response should be investigated in more detail in the future. As described in previous works on biphasic poro-viscoelastic models for soft biological tissues, the (hydraulic) permeability can vary over several orders of magnitude without having a significant impact on the overall response (DiSilvestro and Suh, 2001; Olberding and Suh, 2006). The influence of this parameter could strongly depend on the investigated load cases, which in turn has direct relevance for future simulations and applications of the composite hydrogel. Future work will therefore be devoted to the design and implementation of specific experiments focused on permeability.

**FIGURE 7:**
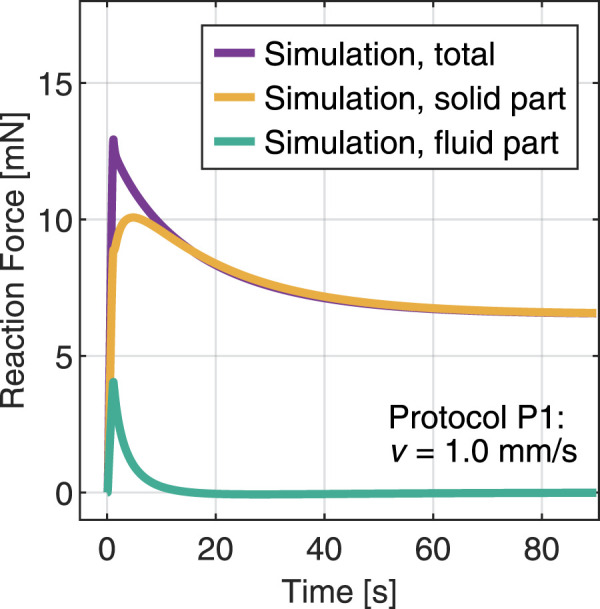
Simulation of an indentation test with protocol P1 and splitting the total reaction force into fluid and solid contributions.

The model proposed here treats the hydrogel as a biphasic material, assuming full saturation conditions. In this framework, the fundamental parameters that control the volumetric relaxation due to fluid flow through the network are the reference porosity, determined *via* the equilibrium water content, and the initial intrinsic permeability (Greiner et al., 2021). The latter particularly determines how easily the interstitial fluid, here deionized water, can move through the porous network. To obtain more insight, Figure 7 displays the splitting of the reaction force into individual solid and fluid contributions for the simulated indentation protocol P1 with the material parameters fitted based on protocol P2. Initially, the pore fluid takes a substantial part of the load. Considering the very large porosity determined for this hydrogel (≈96%) , it seems reasonable to observe such a relevant contribution. However, the fluid part disappears quite quickly after about 10 s. After this time, the solid matrix entirely carries the applied load, indicating that the fluid is flowing rapidly through the region of interest. In contrast to viscous relaxation, the area of indentation affects the characteristic relaxation time within a porous medium (Su et al., 2023) because it changes the amount and distance that the fluid has to move through the solid matrix. The relatively high value of intrinsic permeability in combination with small volumetric forces can explain the marginal role that porous relaxation plays for the given loading conditions. In the future, a differentiation of the mechanical tests, by tuning length and timescales of the experiments, could lead to a better understanding of the separation between fluid-related and viscous relaxation.

## Data Availability

The raw data supporting the conclusion of this article will be made available by the authors, without undue reservation.
