# Supplementary material for: Poro-viscoelastic material parameter identification of brain tissue-mimicking hydrogels
Source: Front Bioeng Biotechnol. 2023 Apr 10;11:1143304. doi: 10.3389/fbioe.2023.1143304 (PMC10123293; doi:10.3389/fbioe.2023.1143304)
Supplement: Supplementary file 1 [file DataSheet1.pdf]

SUPPLEMENTARY MATERIAL

3D-Printed Sample Stage

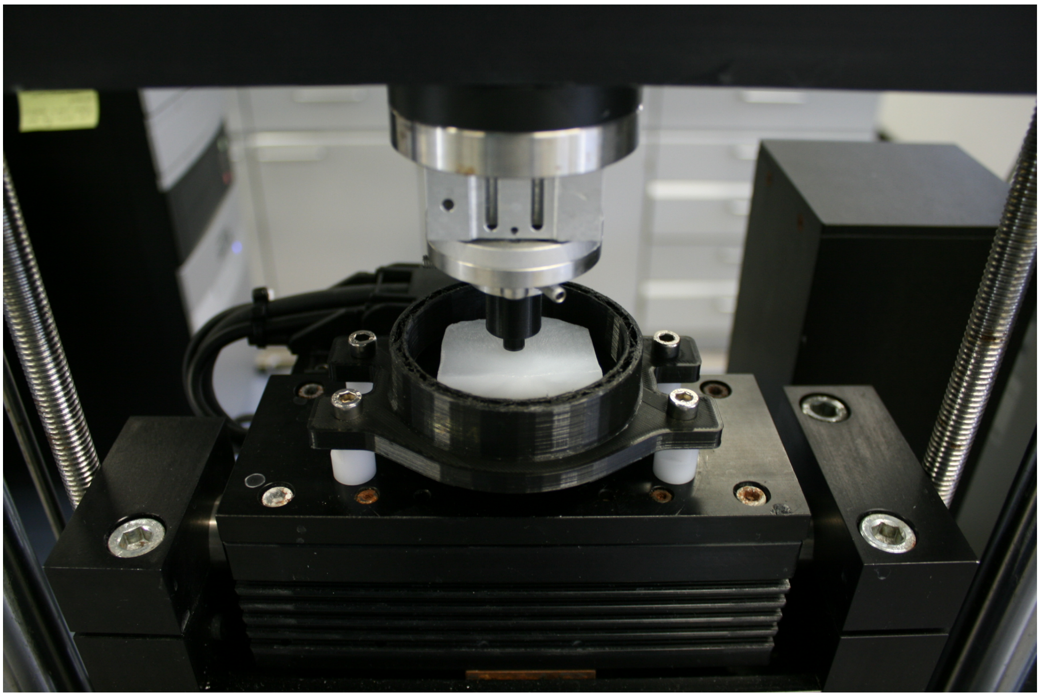

Figure 1. Supplementary figure to demonstrate the entire experimental setup with a new sample stage and new indenter (this setup has not been used for indentation testing before).

Data Processing

The time steps across the measurements were not uniformly distributed in the raw data sets and were therefore standardized in a post-processing step. In a first step, a sampling grid was introduced. Figure 2A shows two exemplary data sets after the introduction of the sampling grid. The requirements for this grid were that it should definitely be finer than the smallest time interval occurring in the data set. Otherwise, the resolution of the data would have been artificially and unnecessarily reduced. Next, a linear interpolation algorithm from one data point to the next was applied. A new data point was placed where the sampling grid intersected the interpolation line (see Figure 2B,C). Thus, in ascending order, a new point was computed at each node of the grid. Since the sampling grid was chosen to be identical for each data set, a synchronized data set could be derived from the raw data (see Figure 2D). A synchronization was performed for the recorded forces and for the recorded displacement data. For each test (P1 and P2) with each material (brain and hydrogel), 10 samples were tested. As a measure of the central tendency of the experimental data, we computed the median from the new, equidistant data and used it for further numerical processing. For demonstration purposes on the two datasets, the data processing is demonstrated with mean and standard deviation in Figure 2E,F. The spread of the experimental data in the main work was derived by computing the interquartile range (IQR) at each of the grid points. The IQR represents the spread of the middle 50% of the data around the median. The experimental data were post-processed in MATLAB (The MathWorks, Inc., Natick, United States).

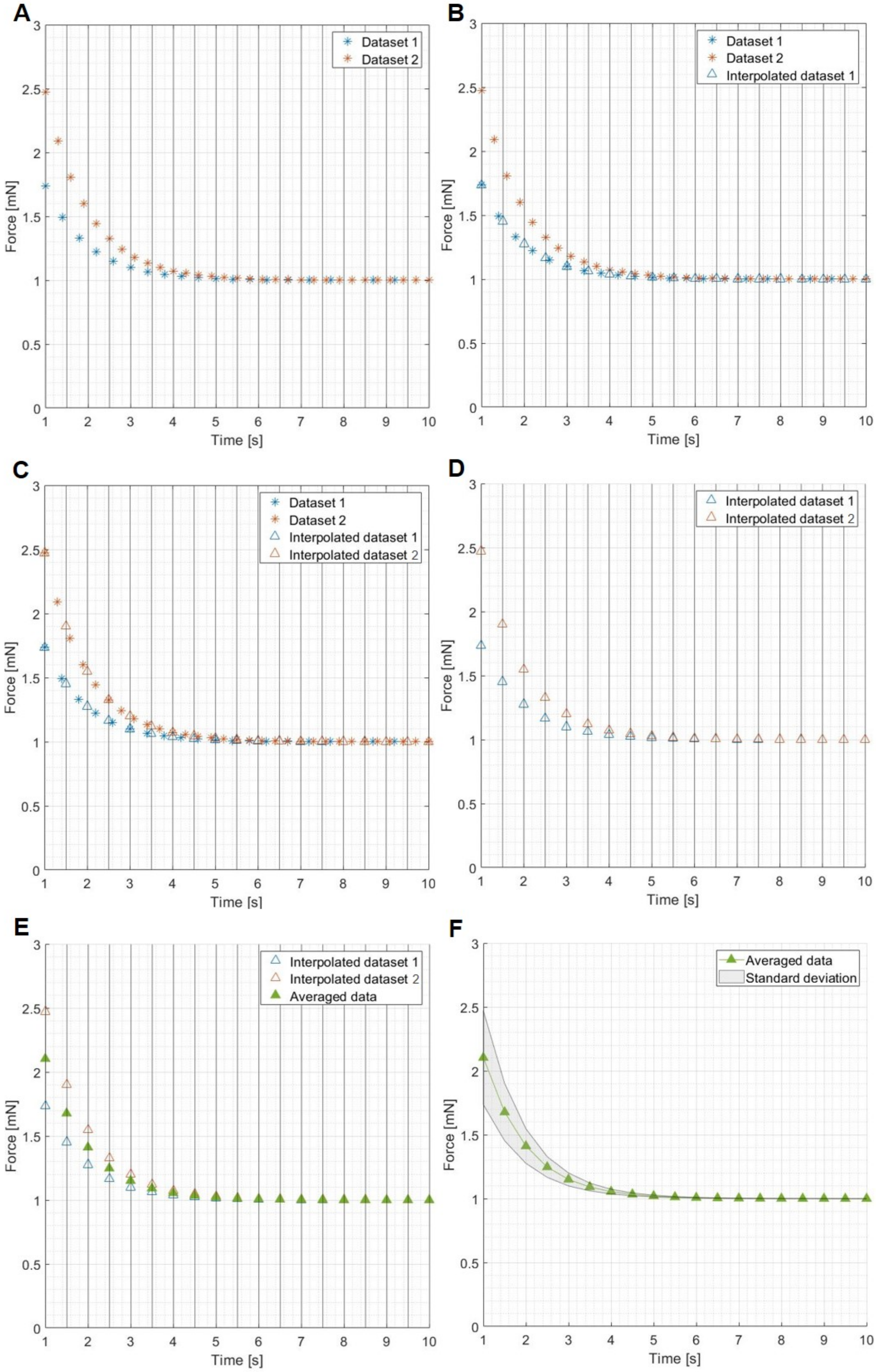

Figure 2. Supplemental figure demonstrating post-processing of the experimental raw data.

Extended Holding Time Shows Non-Relaxed Forces After 90 s

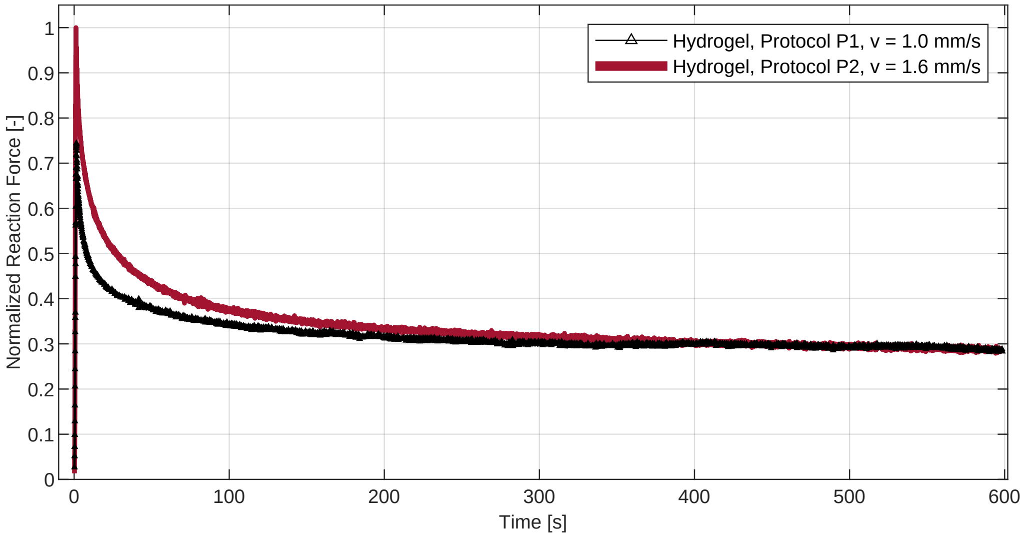

Figure 3. Supplemental figure to demonstrate that the same hydrogel sample achieves the same equilibrium force when the holding time in the indentation is increased. The extended measurement also explains why we do not capture this behavior with the initially introduced protocols with 90 s holding time.
